# Impacts of the New Worldwide Light-Duty Test Procedure on Technology Effectiveness and China’s Passenger Vehicle Fuel Consumption Regulations

**DOI:** 10.3390/ijerph18063199

**Published:** 2021-03-19

**Authors:** Kangda Chen, Fuquan Zhao, Xinglong Liu, Han Hao, Zongwei Liu

**Affiliations:** 1 State Key Laboratory of Automotive Safety and Energy, Tsinghua University, Beijing 100084, China; ckd16@mails.tsinghua.edu.cn (K.C.); zhaofuquan@tsinghua.edu.cn (F.Z.); lxl19@mails.tsinghua.edu.cn (X.L.); hao@tsinghua.edu.cn (H.H.); 2 Tsinghua Automotive Strategy Research Institute, Tsinghua University, Beijing 100084, China; 3 China Automotive Energy Research Center, Tsinghua University, Beijing 100084, China; 4 Sloan Automotive Laboratory, Massachusetts Institute of Technology, Cambridge, MA 02139, USA

**Keywords:** China, CAFC regulations, WLTP, NEDC, fuel consumption

## Abstract

As a main measure to promote the development of China’s energy–saving and new energy vehicles, the Phase V fuel consumption regulation is dramatically different from the past four phases, especially in the test procedure, moving from the New European Driving Cycle (NEDC) to the worldwide harmonized light duty test cycle (WLTC) and corresponding test procedure (WLTP). The switch of test procedure will not only affect the effectiveness of technologies but also change the fuel consumption target of the industry. However, few studies have systematically investigated the impacts of the new WLTP on the Chinese market. This study establishes a “technology–vehicle–fleet” bottom–up framework to estimate the impacts of test procedure switching on technology effectiveness and regulation stringency. The results show that due to the WLTP being closer to the real driving condition and more stringent, almost all baseline vehicles in the WLTP have higher fuel consumption than that in the NEDC, and diesel vehicles are slightly more impacted than gasoline vehicles. In addition, the impacts are increased with the strengthening of electrification, where the fuel consumption of plug–in hybrid electric vehicles (PHEVs) and range-extended electric vehicles (REEVs) in the WLTP are about 6% higher than that in the NEDC. Engine technologies that gain higher effects in low load conditions, such as turbocharging and downsizing, fuel stratified injection (FSI), lean–burn, and variable valve timing (VVT), are faced with deterioration in the WLTP. Among these, the effect of turbocharging and downsizing shows a maximum decline of 8.5%. The variable compression ratio (VCR) and stoichiometric gasoline direct injection (SGDI) are among the few technologies that benefited from procedure switching, with an average improvement of 1.6% and 0.2% respectively. Except for multi–speed transmissions, which have improvement effects in the WLTP, all automatic transmissions are faced with decreases. From the perspective of the whole fleet and national regulation target, the average fuel consumption in the WLTP will increase by about 7.5% in 2025 compared to 4 L/100 km in the NEDC. According to the current planning of the Chinese government, the fuel consumption target of Phase V is set at 4.6 L/100 km in 2025, which is equivalent to loosening the stringency by 0.3 L/100 km. In Phase VI, the target of 3.2 L/100 km is maintained, which is 30.4% stricter than that of Phase V, and the annual compound tightening rate reaches 7.5%. This means that automakers need to launch their product planning as soon as possible and expand the technology bandwidth to comply with the Phase VI fuel consumption regulation, and the government should evaluate the technical feasibility before determining the evaluation methods and targets of the next phase.

## 1. Introduction

**Figure 1:**
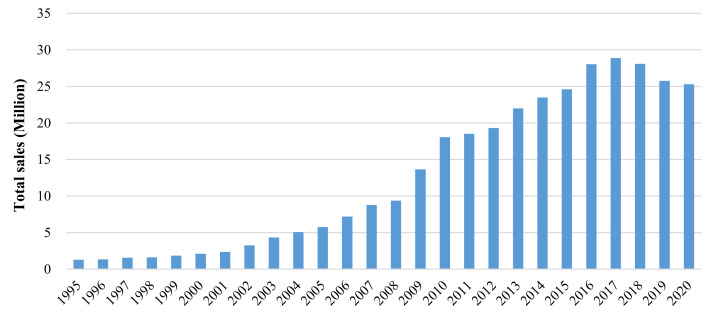
Total automobile sales in China from 1995 to 2020 [3,4].

The Chinese automobile market has achieved dramatic development since 2000. From the perspective of market volume, the market ranks first in the world, accounting for about 28.2% of global automobile sales volume [1], exceeding 20 million for 8 consecutive years since 2013. The automobile production volume and sales volume respectively declined to 25.23 million and 25.31 million in 2020, with year-on-year decreases of 2% and 1.9% [2], due to the impacts of reduced economic growth and the Covid-19 epidemic, but the market is expected to rebound in the coming years. Figure 1 presents the annual sales of automobiles in China from 1995 to 2020.

However, with its rapid development, China is also facing severe energy and environmental problems [5,6,7]. China’s external dependence on oil has continually exceeded the 50% international safety warning line since 2009. In 2011, the number exceeded that of the United States for the first time, reaching 55.2%, and it reached 70.8% in 2019 [6,8], far exceeding the safety warning level. Meanwhile, influenced by the explosion of the automobile market, China has replaced the United States as the world’s largest carbon dioxide emitter since 2006, accounting for 27.6% of global carbon emissions [9]. At the Paris Climate Summit in 2015, China proposed the goal of reducing carbon emissions per gross domestic product (GDP) by 60–65% in 2030, compared with 2005 [10]. After that, at the 75th United Nations General Assembly in 2020, China further announced the goal of striving to reach the peak of carbon emissions before 2030 and achieve carbon neutrality before 2060 [11].

To accelerate the cultivation and development of energy–saving and new energy vehicles, reduce the fuel consumption of vehicles, and relieve the pressure on energy and the environment, the Chinese government has issued and implemented five stages of fuel consumption regulations since 2005, as shown in Table 1. The Phase I and Phase II national fuel consumption limits of passenger vehicles were implemented in 2005 and 2008, respectively [12]. The Phase III regulation was implemented in 2012 [13], which introduced the corporate average fuel consumption (CAFC) evaluation system based on the single-vehicle limit requirements, and automakers could plan their products at their option under the compliance of CAFC regulation. In 2016, China implemented the Phase IV standard, which further tightened the single-vehicle limit and reduced the CAFC targets to 5.0 L/100 km in 2020 [14,15].

Subsequently, China officially issued the Phase V regulations in 2019, which will be implemented since 2021, aiming to further reduce the average fuel consumption to 4.0 L/100 km (the NEDC test) in 2025 [17,18] and promote the market share of new energy vehicles to more than 20% [19,20]. Different from the past four phases regulations, the Phase V fuel consumption regulation replaces the new European driving cycle (NEDC) with the worldwide harmonized light duty test cycle (WLTC) and test procedure (WLTP), which poses great impacts on the effectiveness of energy–saving and new energy vehicle technologies [21,22,23,24], as well as the national technology roadmap and fuel consumption targets.

**Figure 2:**
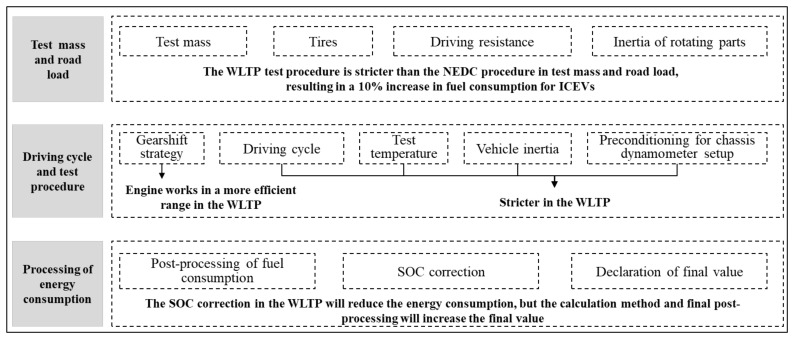
Summary of the NEDC and WLTP test procedure comparisons.

As shown in Figure 2, the WLTP test procedure is different from the NEDC test procedure mainly in three aspects: test mass and road load, driving cycle and test procedure, and post-processing of results [21]. From the perspective of test mass, the vehicle test mass in the NEDC method only includes the benchmark mass, which is curb weight plus 100 kg [22]. In the WLTP, the test mass not only includes the benchmark mass but also includes the mass of optional equipment and representative vehicle load. The total test mass in the WLTP is larger than that of the NEDC procedure [23,24]. In terms of tire selection and tire pressure requirements, the required coefficient of tire rolling resistance is also larger in the WLTP. As for driving resistance, the road load coefficient of the NEDC is lower than that of the WLTP. In addition, the inertia of rotating parts is not taken into account in the NEDC test, which also leads to the driving resistance of the WLTP being higher than that of the NEDC.

**Figure 3:**
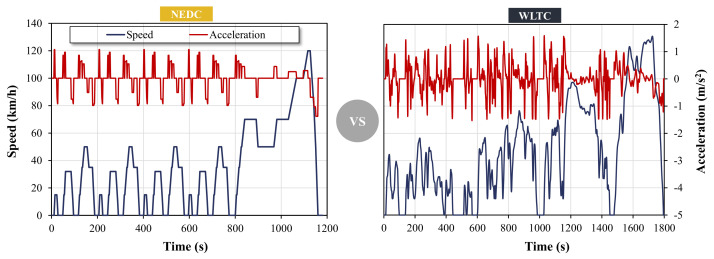
Comparisons of the NEDC and WLTC driving cycles [27].

From the perspective of the driving cycle, the acceleration and deceleration conditions are more frequent in the WLTC, and the maximum speed, as well as the average speed in the WLTC are also larger than that in the NEDC, which increases the fuel consumption of vehicles in the WLTC. In addition, the proportion of idle conditions decrease by half and the constant speed conditions decrease by 36.6%, which directly weakens the effect of hybrid electric technologies. Also, the required starting temperature of the engine has dropped from 23 °C in the NEDC to 14 °C in the WLTP [25]. However, the gearshift strategy in the WLTP test is more flexible, which keeps the engine working in a more efficient area and reduces fuel consumption [26]. Figure 3 depicts the relative speed and acceleration profiles over time of the NEDC and WLTC cycles.

In the post-processing of final energy consumption, more details are considered to modify the fuel consumption in charge depleting (CD) and charge sustaining (CS) modes of PHEVs, for example, the state of charge (SOC) correction in CS mode. In addition, considering the difference between the declared value and the laboratory testing value, different calculation methods are adopted to determine the final type-approved fuel consumption, which also results in a 5% higher number in the WLTP [28].

Since the European Union has replaced the NEDC test procedure with the WLTP test procedure for fuel consumption and emission assessment of light vehicles from 2017, there is extensive research on the comparisons between the NEDC test and the WLTP test, which shows that the WLTC is closer to the real driving condition and more stringent than the NEDC. Marotta et al. tested 21 vehicles both in the NEDC and WLTP and then calculated the WLTP/NEDC emission ratio of different pollutants. The results showed that the road load and test mass have greater influence on the result of carbon emission than the driving cycle. In addition, due to the limited peak power, small vehicles with curb weight less than 1100 kg emit more carbon dioxide in the WLTP than that of the NEDC [24]. Pavlovic et al. tested 20 gasoline vehicles and 11 diesel vehicles in the WLTP and NEDC respectively, and the results showed that with the change in time duration (increasing from 1180 s in the NEDC to 1800 s in the WLTC), test distance (increasing from 11.03 km in the NEDC to 23.27 km in the WLTC), test mass, average speed, acceleration and deceleration, idle duration, and so on, under the best and worst scenarios, carbon emissions in the WLTP test are 1% and 11% higher than that of the NEDC test, and the energy consumption is 26% and 44% higher than that of the NEDC cycle, respectively. In addition, diesel vehicles are more affected by the switching of test procedure [21,28]. Tsiakmakis et al. simulated the test of vehicles with different powertrains in two test cycles based on the PyCSIS model and studied the impacts on carbon emissions. The results showed that almost all vehicles have higher carbon emissions in the WLTP than in the NEDC test. The weighted average WLTP/NEDC emission ratio of the whole European fleet is 1.21, which means the introduction of the new cycle increased the tested fleet’s carbon emissions value by nearly 21%. In addition, with the increase of vehicle carbon emissions, the WLTP/NEDC ratio gradually decreases, that is, the impacts on large vehicles are smaller than that of small vehicles. In terms of different powertrains, test cycle switching has a similar impact on gasoline cars and diesel cars. The WLTP/NEDC ratios of battery electric vehicles (BEVs), fuel cell vehicles (FCVs), and hybrid electric vehicles (HEVs) are larger than that of traditional vehicles, which indicates that the test procedure switching poses a greater impact on electric vehicles [29,30]. Mock et al. compared the similarities and differences between the two cycles, identified the main factors, and quantified the impacts. The results showed that the changes in test mass increase the final carbon emissions and fuel consumption by 3.5%. The changes in the driving cycle and the fleet structure increase the fuel consumption and carbon emissions by 2.1% and the engine starting temperature changes bring an average 1.9% increase. Generally, the impact of test procedure switching on the carbon emission target for 2020 is 7.5% [24].

However, few studies have systematically investigated the impacts of the new WLTP test procedure on the Chinese fuel consumption regulation, market, and technology effectiveness. On the one hand, China’s Phase V fuel consumption regulation was newly issued. The relevant research has not yet caught up. On the other hand, most of the former research aims at the European markets rather than the Chinese market, and almost all these studies focus on the vehicle level rather than the technical level.

To fill these research gaps, this study establishes a “technology–vehicle–fleet” multi-scale and bottom–up research framework to estimate the impacts of test procedure switching on technology effectiveness and regulation stringency. First, extensive technologies are identified, screened, and unified for the Chinese market, and finally, the effectiveness of 10 powertrains and 64 technologies are determined and investigated based on the Passenger car and Heavy duty Emission Model (PHEM) [31] and multi-source data. In addition, the stringency of national CAFC regulations is analyzed from the perspective of the whole fleet, in order to provide implications and quantitative suggestions for the determinations of national fuel consumption targets and technical routes under the new phase of regulations. This study includes evaluations of technology effectiveness, predictions of fleet trends, and an explicit assessment of policy implications. The paper is organized as follows. Section 2 describes the research framework, methodology, key assumptions, and data. Section 3 analyzes and discusses the main results from two aspects of technology and regulation. Section 4 presents the policy implications, proposes suggestions for the government and automakers, and summarizes the whole study.

## 2. Materials and Methods

In this section, the overarching framework of the research is introduced first. Then, a detailed description of the database is given, and then the specific calculation methods are explained, including the definition of the change rate of fuel consumption, technical effect, the change rate of technical effect, and the fleet impact factor.

A bottom–up research framework that is multi-scale and covers the technology level, vehicle level, and fleet level is established in this study. First, extensive technologies from different mainstream markets are identified, screened, and unified for the Chinese market. These include various combustion, electric-drive, and hybrid-drive vehicles, where a total of 10 powertrains and 64 energy–saving and new energy technologies are identified. Then, vehicles with different powertrains and technologies are simulated and investigated based on the PHEM model [31] and multi-source data. The effectiveness of various technologies and the preference of the WLTP are also evaluated. Finally, the stringency of national CAFC regulations are analyzed from the perspective of the whole fleet based on technology effectiveness, and the predictions of fleet structure and technology penetration.

### 2.1. Data and Assumptions

As mentioned above, the impact of the new WLTP test procedure on the fuel consumption of vehicles and the effect of different technologies are extremely complicated and affected by a variety of factors. More accurate results can be gained from real vehicle tests or simulations. The main technical data sources of this study are simulations, and the data are modified further according to other data sources. Ricardo and TU Graz conducted extensive simulations of the impacts of different technologies on the CO2 emissions from different light-duty vehicle segments, powertrain types, and test cycles using the PHEM model [31,32]. Based on the original technical data from Ricardo and TU Graz [31], data calibration, cleaning, and localization are performed through other multi-source data from Joint Research Center (JRC), US National Research Committee (NRC), International Council on Clean Transportation (ICCT), China Automotive Technology & Research Center (CATARC), Society of Automotive Engineers (SAE-China), and enterprise survey [32,33,34]. The baseline passenger vehicles in the Chinese market are divided into four segments: small (A00 and A0), compact (A), midsize (B), and large (C and D). Since the factor measured in the Chinese market is fuel consumption rather than carbon emission, the original carbon emission data are converted to fuel consumption by the ICCT energy consumption conversion model [35].

On the processing of various technologies from different mainstream markets, we identify the concepts and technical principle of all advanced energy–saving and new energy technologies, and we screen and unify all these technologies for the Chinese market, as shown in Table 2. The 4 categories (engine, transmission, vehicle and accessory, electric), as well as the assignments of 64 different technologies, are set according to the technology attributes, which are widely used by the US NRC, Environmental Protection Agency (EPA), and other official institutes.

The market data for China’s passenger vehicles are mainly from the historical and prediction material published by the Chinese government, CATARC, SAE–China, China Passenger Car Association (CPCA), etc. [1,3,4,36,37]. The technology penetration rate data is determined according to the technology pathway selection and optimization model [38,39,40] and the newly issued Energy–saving and New Energy Technology Roadmap 2.0 [41]. Parameters of baseline vehicles are shown in Table 3.

### 2.2. Calculation Methods

The specific calculation methods are explained in this section. Main indicators including the change rate of fuel consumption, technology effectiveness, the change rate of technology effectiveness, and the fleet impact factor, are defined and indicated in Equations (1)–(6). For a certain year within the time frame (2015, 2020, 2025, 2030), we investigate the impact from these indicators.

(1) $$r_{i,j}=\frac{f_{i,j,WLTP}-f_{i,j,NEDC}}{f_{i,j,NEDC}}\times 100\%$$

where *i* represents the vehicle segment, including four types: small, compact, midsize, and large;

*j* represents the powertrain type, including ICE gasoline, ICE diesel, Start–stop, Micro hybrid, Mild hybrid, Strong hybrid, PHEV, REEV, BEV, FCV;

*f_i,j,WLTP_* represents the fuel consumption of vehicle with segment *i*, powertrain *j* in the WLTP test;

*f_i,j,NEDC_* represents the fuel consumption of vehicle with segment *i*, powertrain *j* in the NEDC test;

*r_i,j_* represents the fuel consumption change rate of vehicle with segment *i*, powertrain *j* between two test procedures, which represents the attributes of baseline vehicles. The larger the *r_i,j_*, the higher the test cycle-switching impact on the fuel consumption of the baseline vehicle.

(2) $$r_{i,j,k}=\frac{f_{i,j,k,WLTP}-f_{i,j,k,NEDC}}{f_{i,j,k,NEDC}}\times 100\%$$

where *k* represents the type of advanced energy–saving and new energy technology, including 64 kinds of technologies; and *r_i,j,k_* represents the fuel consumption change rate of vehicle with technology *k*, segment *i*, and powertrain *j* between two test procedures. The larger the r*_i,j,k_*, the higher the test cycle-switching impact on fuel consumption of single vehicle with technology *k*.

(3) $$E_{i,j,k,NEDC}=\frac{f_{i,j,0,NEDC}-f_{i,j,k,NEDC}}{f_{i,j,0,NEDC}}\times 100\%$$

where *E_i,j,k,NEDC_* represents the effectiveness of technology *k* deployed in vehicle with segment *i*, powertrain *j* in the NEDC test. The larger the *E_i,j,k,NEDC_*, the higher the technology effectiveness in the NEDC test.

*f_i,j,0,NEDC_* represents the fuel consumption of the vehicle (segment *i*, powertrain *j*) without technology *k* in the NEDC test;

(4) $$E_{i,j,k,WLTP}=\frac{f_{i,j,0,WLTP}-f_{i,j,k,WLTP}}{f_{i,j,0,WLTP}}\times 100\%$$

where *E_i,j,k,WLTP_* represent the effectiveness of technology *k* deployed in vehicle with segment *i*, powertrain *j* in the WLTP test. The larger the *E_i,j,k,WLTP_*, the higher the technology effectiveness in the WLTP test.

(5) $$\Delta E_{i,j,k}=E_{i,j,k,NEDC}-E_{i,j,k,WLTP}$$

where Δ*E_i,j,k_* represent the effectiveness difference of technology *k* deployed in vehicle with segment *i*, powertrain *j* between two tests. The larger the Δ*E_i,j,k_*, the higher the test cycle-switching impact on effectiveness of single technology.

(6) $$p_{fleet}=\frac{\sum_{i}\sum_{j}\sum_{k}w_{i}\cdot w_{ij}\cdot w_{i,j,k}\cdot r_{i,j,k}\cdot f_{i,j,k,NEDC}}{\sum_{i}\sum_{j}\sum_{k}w_{i}\cdot w_{ij}\cdot w_{i,j,k}\cdot f_{i,j,k,NEDC}}\times 100\%$$

where *w_i_* represents the penetration rate of vehicle with segment *i*;

*wi,j* represents the penetration rate of vehicle with segment *i*, powertrain *j*;

*w_i,j,k_* represent the penetration rate of technology *k* deployed in vehicle with segment *i*, powertrain *j*;

*P_fleet_* represents the impact factor of test procedure switching on the fleet.

The effectiveness changes of various technologies will pose an impact on the fuel consumption of vehicles. Combined with the penetrations of vehicles and technologies, the final change of fleet fuel consumption could be obtained. *P_fleet_* represents the change rate of fleet fuel consumption caused by the test cycle switching. In addition, the larger the *P_fleet_*, the higher the test cycle switching impact on fuel consumption of the whole fleet.

## 3. Results and Discussion

As mentioned above, an important difference between China’s Phase V fuel consumption and the previous four phase regulations is the change of the test procedure, from NEDC to WLTC, where new indicators are based on the WLTP test. The new test procedure will pose a significant impact on vehicle fuel consumption, regulations, and technology routes. In this section, the effectiveness of 10 powertrains and 64 technologies are investigated, and the stringency of national CAFC regulations is analyzed from the perspective of the whole fleet, in order to provide implications and quantitative suggestions for the determinations of national fuel consumption targets and technical routes under the new phase of regulations.

### 3.1. Impacts on the Effectiveness of Energy–Saving and New Energy Technologies

**Figure 4:**
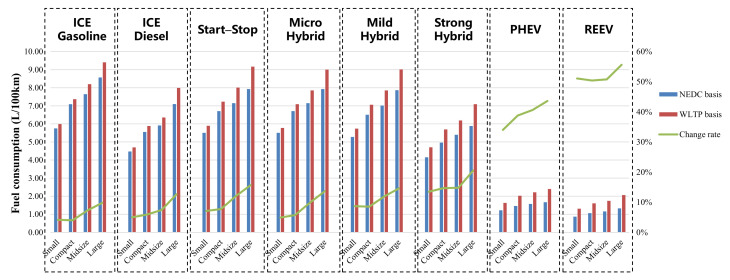
Fuel consumption of the baseline vehicles with different powertrains.

The baseline vehicles in this study are vehicles in four segments with 10 different powertrains. Since the fuel consumption of BEVs and FCVs is calculated as 0 in the Phase V regulation, the change of test procedures has no effect on the fuel consumption. So, they are not presented in this part. The fuel consumption of different baseline vehicles is shown in Figure 4.

It can be seen from the simulation results of fuel consumption of baseline vehicles that the comprehensive fuel consumption of almost all vehicles, whether gasoline or diesel, in the WLTP is higher than that in the NEDC. The fuel consumption difference between different test procedures gradually increases with the increase of vehicle mass. Compared with gasoline vehicles, diesel vehicles are more impacted by the transition of test procedure from NEDC to WLTP, although diesel vehicles have lower fuel consumption. From the perspective of electrification degree, vehicles with higher electrification are more effected by the transition. Among them, PHEVs and REEVs are the most affected, mainly due to the significant change of driving cycle and the calculation method of energy consumption. However, the fuel economy rankings have not been changed; vehicles with a higher electrification degree still have higher fuel economy, no matter in which test procedure.

**Figure 5:**
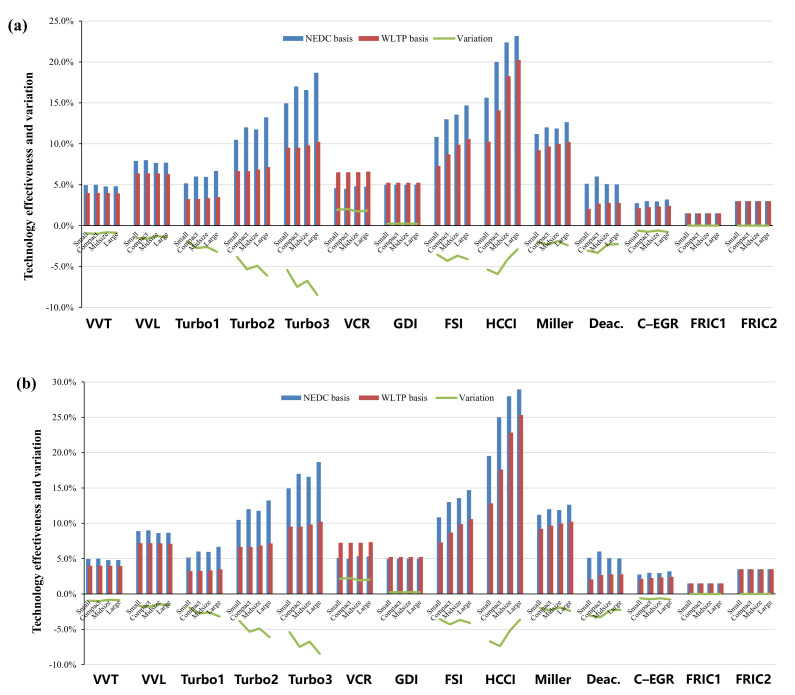
(**a**,**b**): Comparisons of advanced gasoline engine technologies in 2025 and 2030. Notes. (1) Turbo1, Turbo2, and Turbo3 represent mild downsizing (15% cylinder content reduction), medium downsizing (30% cylinder content reduction), and strong downsizing (≥45% cylinder content reduction) respectively. (2) FRIC1 and FRIC2 represent a 20% and 40% reduction in engine friction respectively [31].

**Figure 6:**
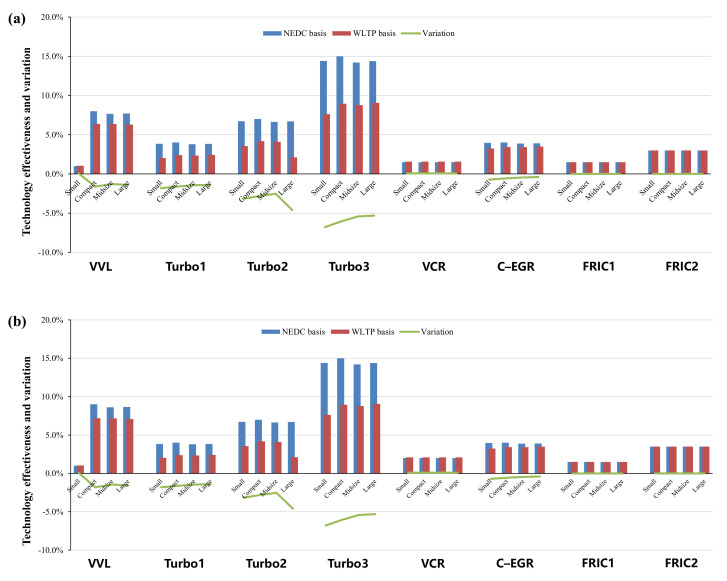
(**a**,**b**): Comparisons of advanced diesel engine technologies in 2025 and 2030.

**Figure 7:**
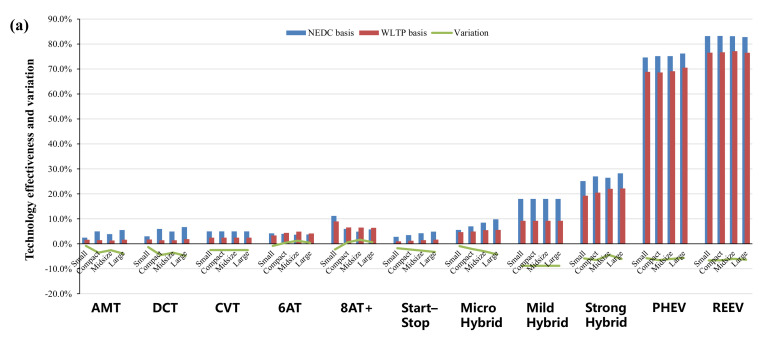

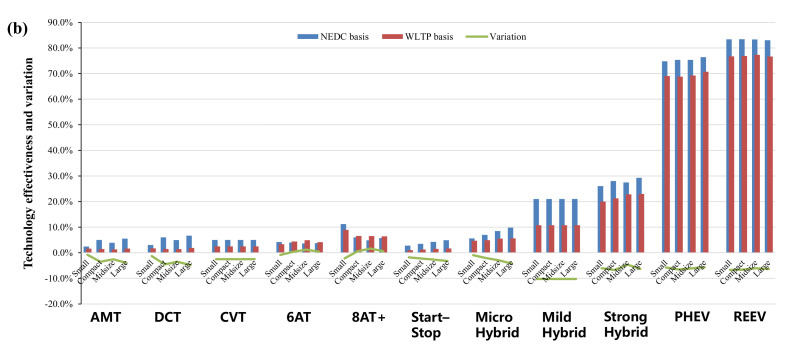
(**a**,**b**): Comparisons of transmission and electric technologies in 2025 and 2030.

**Figure 8:**
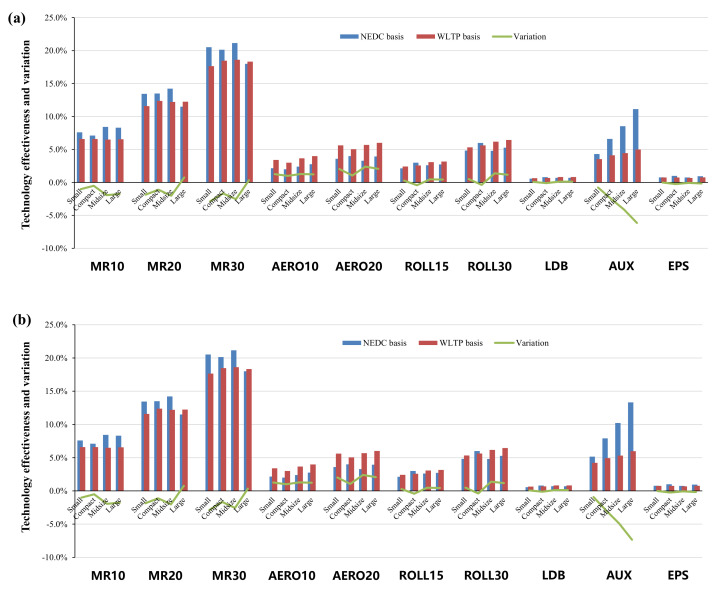
(**a**,**b**): Comparisons of vehicle and accessory technologies in 2025 and 2030. Notes. (1) MR10, MR20, and MR30 represent 10%, 20%, and 30% mass reduction from the whole vehicle respectively. (2) AERO10 and AERO20 represent 10% and 20% reduction in air resistance coefficient respectively. (3) ROLL15 and ROLL30 represent 15% and 30% reduction in rolling resistance [31].

Specifically, in terms of the energy–saving effects of various advanced technologies in NEDC and WLTP, Figure 5, Figure 6, Figure 7 and Figure 8 show the effectiveness of 64 technologies in four categories, including advanced gasoline engine technology, advanced diesel engine technology, transmission and electric technology, and vehicle and accessory technology under different test cycles and at different time points.

As shown in Figure 5a,b, among all gasoline technologies, homogeneous charge compression ignition (HCCI) has the greatest energy–saving potential. In 2030, the highest effectiveness in the WLTP of HCCI can reach about 25%, followed by turbocharging and the Miller cycle, which reach 16.6% and 12.6%, respectively. The effects of technologies that could gain more obvious effects under low load conditions (turbocharging and downsizing, cylinder deactivation, FSI, HCCI, VVT) are generally deteriorated in the WLTP test. Of these, the turbocharging and downsizing technology is facing the largest impact, with a maximum decline of 8.5%. As for cylinder deactivation, FSI, HCCI, and VVT technologies, the decline range is respectively 1.9–3.3%, 2.5–4.3%, 0.8–7.4%, and 0.5–1% for different segments. The VCR and SGDI technologies are the few technologies that benefit from the WLTP test due to the higher load variation, where the effectiveness of VCR increases by 1.6% and that of SGDI increases by 0.2% on average. In addition, there is no effect on the friction reduction technology of gasoline engines.

In terms of advanced diesel engine technologies, the effects of turbocharging and downsizing also drop off significantly, with a decrease of 1.4–6.8%, as shown in Figure 6. The effectiveness of VCR increases by about 0.1% in the WLTP. The friction reduction technology of diesel engines is also almost not affected by the change of test procedure.

With regard to the transmission technologies, the multi–speed technology has more obvious advantages under the WLTP test which contains higher average speed and more transient conditions, as shown in Figure 7. However, the effectiveness of other automatic transmissions decrease in the WLTP test.

In terms of electric technologies, also shown in Figure 7, compared to the NEDC cycle, the percentage of idle conditions in the WLTP driving cycle has fallen sharply. Thus, the effects of hybrid electric technologies, such as start–stop and mild hybrid technologies, are significantly lower than that in the NEDC test. As for PHEVs and REEVs, the fuel-saving effects of PHEVs and REEVs under the WLTP test procedure is about 6% lower than that under the NEDC procedure due to the change of driving cycle and energy consumption calculation method.

We also examined vehicle and accessory technologies, where a high level of lightweight technology (20–30% lightweight rate) applied to large vehicles under the WLTP show increased effectiveness compared with that of the NEDC, while the energy–saving effect is decreased for other technologies. Due to the higher speed in the WLTP, the effectiveness of low wind resistance, low rolling resistance, and low drag braking is improved by about 1.2–2.4%, 0.4–1.4%, and 0.1%, respectively. The auxiliary systems efficiency improvement (AUX) and electric power steering (EPS) are faced with decreased effectiveness, and the deterioration of AUX is more obvious with a range of 0.8–6.1%, where the larger the model, the more serious the deterioration will be.

### 3.2. Impacts on the Stringency of National Fuel Consumption Regulations

**Figure 9:**
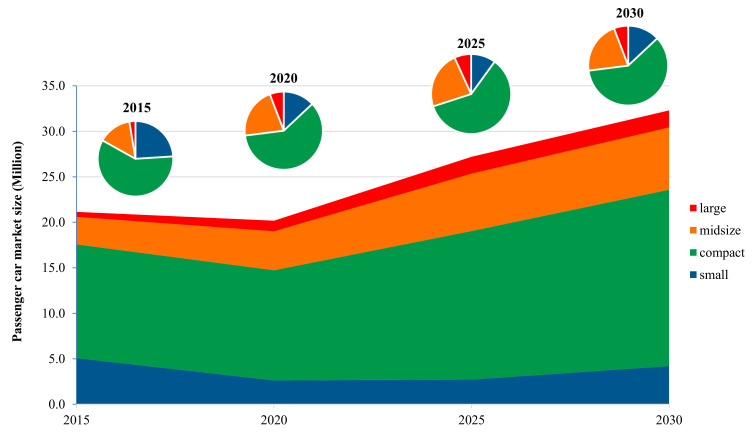
Structure development of China’s passenger car market.

**Figure 10:**
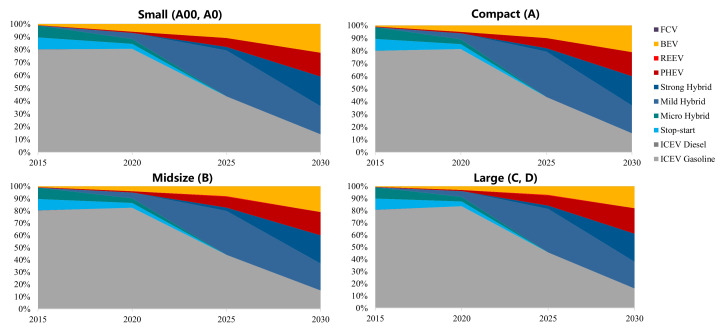
Penetration of different powertrains in the Chinese market.

In order to study the impact of the new WLTP test procedure on the fleet fuel consumption, this study combined the calculation results of the technology route selection model and the planning of national technology roadmap 2.0 to forecast the market structure and market share of different powertrains and technologies in China, as shown in Figure 9 and Figure 10. In terms of the development of the vehicle segment in the Chinese market, the trend of large passenger cars is obvious in recent years. The average curb weight of passenger vehicles increases by about 1.5% per year since 2016 [42,43,44]. In 2015, small, compact, midsize, and large passenger vehicles account for 24%, 59.1%, 14.3%, and 2.6% of the whole market, respectively. The percentages become 13%, 60%, 21.2%, and 5.8% in 2020, where the relative number of small cars that is projected decreases, while larger cars see a considerable growth. The structure of the passenger car market will be mainly driven by the preference of Chinese consumers and the demand for energy–saving and emission reduction in the future. It is predicted that the proportion of SUV sales in China will gradually reach the saturation point, and then, with the trend of electrification and the requirements of energy–saving and emission reduction, passenger cars will present a trend of miniaturization [45].

From the perspective of powertrains, for the continuously tightening regulations, hybrid technology, especially integrated starter generator (ISG) technology will be substantially deployed. The penetration rate of electric powertrains in small vehicles is expected to see the fastest and most rapid increase compared to other segment vehicles. Due to the higher requirements for driving range, PHEV powertrains will be deployed in midsize and large vehicles with a relatively higher penetration than other segments. The penetration of BEV is approximately 10% in 2025, by which time the total market share of new energy vehicles will be close to 20%.

**Figure 11:**
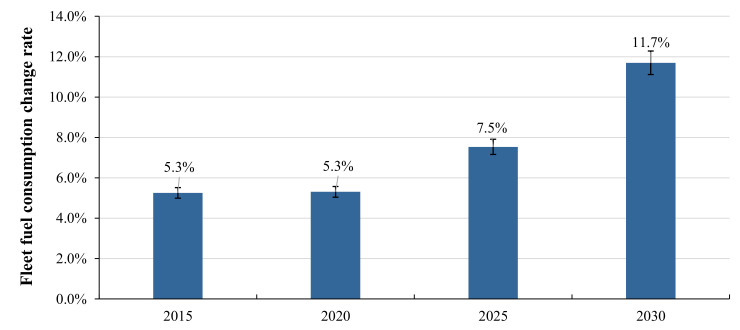
Change rate of China’s fleet fuel consumption under different test procedures. Notes. (1) The error bars represent the value range under 5% margin of error.

According to the bottom–up research framework of “technology–vehicle–fleet”, the final impacts on the whole Chinese passenger car fleet can be gained based on the analyses of technology effectiveness, fleet structure, and technology penetration. The results show that the effectiveness of most technologies will drop off, which directly increases the difficulty of compliance with national fuel consumption regulation. The average fleet fuel consumption in the WLTP test in 2025 will increase by about 7.5% relative to that in the NEDC test, and the national target should be increased from 4 L/100 km in the NEDC to 4.3 L/100 km in the WLTP if keeps the same stringency in different test procedures. If the WLTP is still used in the Phase VI regulation, the average fleet fuel consumption in 2030 will increase by about 11.7%, and the national fuel consumption target value will be increased from 3.2 to 3.6 L/100 km if keeps the same stringency, as shown in Figure 11.

The average reduction rate of the national fuel consumption target in each phase is 20%. According to China’s newly issued energy–saving and new energy technology roadmap 2.0 and the Phase V fuel consumption regulation, China will adjust the national average fuel consumption target to 4.6 L/100 km in 2025. Considering the switching difficulty of enterprise products to different driving cycles, the national average fuel consumption target is relaxed 0.3 L/100 km compared to the numerical value in the NEDC. However, the government is expected to keep the target value of 3.2 L/100 km in 2030, which means that from the 4.6 L/100 km in the Phase V to the 3.2 L/100 km in the Phase VI, the target has been tightened by 30.4% with an average annual compound tightened rate of 7.5%. This is far above the international average tightened rates of 3–6% [46,47,48]. Therefore, from the perspective of the stringency of regulation, the difficulty of complying with China’s Phase V fuel consumption regulation will be lower than expected, but the Phase VI regulation for 2025–2030 will be a great challenge for automakers, and this will bring great pressure on the planning of products and technologies.

## 4. Conclusions

As a main measure to promote the technology development of China’s energy–saving and new energy vehicles, alleviate the pressure on energy and the environment, and drive the transformation of the national energy structure, the CAFC regulation has played an important role in the past ten years and will continue to pose a dramatic impact on the development of the Chinese automobile market. Different from the past four phases, great changes are set in the Chinese Phase V fuel consumption regulation from two aspects: the test procedure and the evaluation system of the regulatory target value. Among them, the switch of test procedure not only has an impact on the effectiveness of various energy–saving and new energy technologies but also affects the setting of national targets and the selection of industry technology routes. However, due to China’s Phase V fuel consumption regulation being newly issued, few studies have systematically investigated the impacts of the new WLTP test procedures on the Chinese fuel consumption regulation, market, and technologies. Most of the research is aimed at the European markets, and almost all these studies only involve the vehicle level rather than the technology level.

This paper focuses on the research gap and establishes a “technology–vehicle–fleet” multi-scale and bottom–up research framework to estimate the impacts of test procedure switching on technology effectiveness and regulation stringency. First, the technology effectiveness of 10 powertrains and 64 technologies are investigated based on the PHEM simulation model and the ICCT energy consumption conversion model. In addition, the compliance of national CAFC regulations is analyzed from the perspective of the whole fleet to provide implications and suggestions for the selection of national targets and technical routes under the new phase of regulations.

The results show that almost all baseline vehicles under the WLTP test have higher fuel consumption than the NEDC procedure since the WLTP is closer to the real road condition and more stringent than the NEDC. In addition, it can be seen from the comparisons between gasoline vehicles and diesel vehicles that the influence on diesel vehicles is slightly greater than that of gasoline vehicles. By comparing different powertrains, it can be seen that the overall trend is that with the enhancement of electrification, the test procedure impacts on the fuel consumption is greater.

In terms of advanced gasoline engines, the effects of technologies with more obvious effects under low load conditions (turbocharging and downsizing, cylinder deactivation, FSI, HCCI, VVT) are generally deteriorated, among which turbocharging and downsizing had the largest impact, with a maximum decline of 8.5%. In addition, VCR and SGDI technologies are the few technologies that benefit from the WLTP cycle due to the greater load variation, where VCR technology increases by 1.6% and SGDI technology increases by 0.2% on average. The gasoline engine friction reduction technology effect is not affected by the change of driving cycle. The same trend also occurs in the advanced diesel engines, and the effect of turbocharging and downsizing also decreased significantly, with a decrease of 1.4–6.8%. The effectiveness of VCR increases by about 0.1% under the WLTP cycle, and the friction reduction technology of diesel engines is almost not affected by the change of test procedure. As for the transmission technology, the multi–speed technology has more obvious advantages under the WLTP. However, the effectiveness of other automatic transmissions decreases in the WLTP test.

With regard to electric technologies, compared to the NEDC driving cycle, the percentage of idle durations in the WLTP driving cycle has fallen sharply; thus, the effects of hybrid electric technologies such as start–stop are significantly lower than that in the NEDC. As for PHEVs and REEVs, the average fuel-saving effect of PHEVs and REEVs under the WLTP is about 6% lower than that under the NEDC procedure due to the change of driving cycle and energy consumption calculation method. In terms of vehicle and accessory technologies, due to the higher speed in the WLTP, the effectiveness of low wind resistance, low rolling resistance, and low drag braking is improved by about 1.2–2.4%, 0.4–1.4%, and 0.1% respectively. The AUX and EPS are faced with decreases in energy-saving, and the deterioration of AUX is more obvious; where the larger the segment is, the more serious the deterioration will be.

From the perspective of the whole Chinese market and the targets of national regulations, the test procedure in Phase V will be changed from NEDC to WLTP, which will be closer to the real driving condition and increase the national average fuel consumption by about 7.5% in 2025. Compared with the target of 4 L/100 km under the NEDC, the target value should be increased from 4 to 4.3 L/100 km. If the WLTP is still maintained in Phase VI, the average fuel consumption in 2030 will increase by about 11.7%, and the national target value should be increased from 3.2 L/100 km in the NEDC to 3.6 L/100 km in the WLTP. According to China’s technology roadmap for energy–saving and new energy vehicles 2.0 and the Phase V regulation that has been newly issued, the national average fuel consumption target was adjusted to 4.6 L/100 km in 2025 and retains the target value of 3.2 L/100 km in 2030. From the perspective of the stringency, it means that the national target is relaxed by 0.3 L/100 km considering the switching difficulties from the NEDC to the WLTP. In Phase VI, the target is 3.2 L/100 km, which is 30.4% stricter than that of Phase V, and the annual compound tightened rate reaches 7.5%. So, the difficulty of complying with the Phase V fuel consumption regulation in China will be smaller than expected, but the Phase VI fuel consumption regulation for 2030 will be a great challenge for automakers.

Therefore, for automakers, on the one hand, preparing for the 2021 test cycle switching, and making full use of technologies that have better effect under the WLTP will help them to comply with the regulation with lower cost and better effect. On the other hand, facing the more stringent Phase VI fuel consumption regulation, automakers need to launch their product planning as soon as possible and expand the technology bandwidth to comply with the regulation. From the perspective of the government, the implementation of the new WLTP test procedure is a big step to improve the accuracy of the whole fuel consumption regulation system. However, according to the stringency calculation, the national fuel consumption target of Phase V is relaxed about by 0.3 L/100 km, while maintaining the 3.2 L/100 km target in Phase VI is equivalent to a tightening of 0.4 L/100 km. Furthermore, the target in Phase VI will be tightened further, and the test procedure will be switched to the China automotive test cycle (CATC). Therefore, the government needs to evaluate the compliance feasibility of national standards based on technology basis and technology potential and also compile the next stage fuel consumption evaluation methods and targets as early as possible.

Based on this study, several topics can be studied further. First, this study focuses on the impacts of the new WLTP on technology effectiveness and fuel consumption regulations. In the future, the test procedure of China’s fuel consumption regulations will further switch to the CATC [49]. Compared to the WLTC, the CATC is also an instantaneous test cycle that is closer to the real driving condition, and it has the same test duration as the WLTC. However, the CATC localized the test cycle and reflected the real driving condition in China further, with a lower average speed, higher idle percentage, and higher acceleration and deceleration percentage. Therefore, technology effectiveness assessment based on the CATC will be important in the next phase. Second, the fuel consumption of BEVs and FCVs is regarded as 0 now in the context of the present CAFC regulations. However, with the increasing concern of carbon emission, there is no doubt that the energy consumption or carbon emissions of BEVs and FCVs will be considered in the regulation. Therefore, the impacts of test procedure switching on the energy consumption of BEVs and FCVs should also be taken into account.

## Figures and Tables

**Table 1:** Phases of China’s passenger vehicle fuel consumption regulations [16].

Phases	Time Frame	Standard	Test Cycle	Description
Phase I	2005.07–2008.01: New type approval vehicle 2006.07–2009.01: Vehicle under manufacturing	GB19578-2004	NEDC	Only the fuel consumption limit for a single vehicle is required; Only for domestic cars
Phase II	2008.01–2012.07: New type approval vehicle 2009.01–2012.07: Vehicle under manufacturing	GB19578-2004	NEDC	Only the fuel limit for a single vehicle is required; Only for domestic cars
Phase III	2012.07–2015.12	GB19578-2004 GB27999-2011	NEDC	The single limit and the ratio of CAFC are both required; Imported cars are included
Phase IV	2016.01–2020.12	GB19578-2014 GB27999-2014	NEDC	The single limit and the ratio of CAFC are both required; Imported cars are included
Phase V	2021.01–2025.12_	GB19578-2021 GB27999-2019	WLTC	The single limit and the ratio of CAFC are both required; Imported cars are included; The evaluation system changed from ladder-type to line-type

**Table 2:** Data and parameters specification.

Category	Description
Data Sources	Ricardo, JRC, ICCT, CATARC, SAE
Data Acquisition Methods	PHEM simulation model, literature research, enterprise survey
Vehicle Segments	Small (A00,A0), Compact (A), Midsize (B), Large (C, D)
Technology	4 Categories: Engine, transmission, vehicle and accessory, electric 10 Powertrains: ICE gasoline, ICE diesel, Start–stop, Micro hybrid, Mild hybrid, Strong hybrid, PHEV, REEV, BEV, FCV 64 Technologies: SGDI, VVT, VVL, HCCI, AMT, CVT, DCT, AERO, HEV, PHEV, REEV, BEV, FCV etc. (This paper only selects some representative technologies to present)
Time Frame	2015, 2020, 2025, 2030
Data Calibration and Correction	Baseline vehicles and technologies from different countries are unified for the Chinese market Immature technologies, or technologies with little effect before 2030, such as steer-by-wire and brake-by-wire, in-wheel motors are eliminated Diesel PHEV, diesel REEV, FCV hybrid, CNG vehicles are eliminated Off-cycle technologies and others not applicable in the Chinese market are not included

**Table 3:** Parameters of baseline vehicles.

Class	Gasoline Vehicles			Diesel Vehicles		
Parameter	Curb Weight (kg)	Footprint (m^2^)	Peak Power (kW)	Curb Weight (kg)	Footprint (m^2^)	Peak Power (kW)
Small	1091	3.6	61	1244	3.7	66
Compact	1380	4.1	91	1510	4.1	91
Midsize	1523	4.3	120	1659	4.3	113
Large	1850	4.6	183	1926	4.7	143

## Abbreviations

AMT	Automated Manual Transmission
AERO	Aerodynamics improvement
AUX	Auxiliary systems efficiency improvement
BEV	Battery Electric Vehicle
CAFC	Corporate Average Fuel Consumption
CAFE	Corporate Average Fuel Economy
CATARC	China Automotive Technology & Research Center
CATC	China Automotive Test Cycle
CD	Charge Depleting
CEGR	Cooled Exhausted Gas Recirculation
CS	Charge Sustaining
CVT	Continuously variable transmission
DCT	Dual Clutch Transmission
DEAC	Cylinder Deactivation
EPS	Electric Power Steering
EPA	Environmental Protection Agency
FCV	Fuel Cell Vehicle
FRIC	Engine Friction Reduction
FSI	Fuel Stratified Injection
HCCI	Homogeneous Charge Compression Ignition
HCR	High Compression Ratio
HEV	Hybrid Electric Vehicle
ICCT	International Council on Clean Transportation
ICEV	Internal Combustion Engine Vehicle
ISG	Integrated Starter Generator
JRC	Joint Research Center
LDB	Low Drag Brakes
MIIT	Ministry of Industry and Information Technology
MR	Mass Reduction
MSRP	Manufacturer’s Suggested Retail Price
NEDC	New European Driving Cycle
NEV	New Energy Vehicle
NHTSA	National Highway Traffic Safety Administration
OEM	Original Equipment Manufacturer
PEV	Plug–in Electric Vehicle
PHEV	Plug–in Hybrid Electric Vehicle
PHEM	Passenger car and Heavy duty Emission Model
REEV	Range-Extended Electric Vehicle
ROLL	Low Rolling Resistance Tires
SAE	Society of Automotive Engineers
SGDI	Stoichiometric Gasoline Direct Injection
SUV	Sport Utility Vehicle
TURBO	Turbocharging and downsizing
VCR	Variable Compression Ratio
VVT	Variable Valve Timing
VVL	Variable Valve Lift
WLTP	Worldwide harmonized Light duty vehicle Test Procedure
WLTC	Worldwide harmonized Light duty vehicle Test Cycle

## References

[1] OICA (2020). Sales of New Vehicles 2005–2019. http://www.oica.net/category/sales-statistics/.

[2] CAAM (2021). Economic Performance of the Automobile Industry in 2020. http://www.caam.org.cn/chn/1/cate_2/con_5232916.html.

[3] CAAM, CATARC, Toyota (2018). Annual Report on Automotive Industry in China (2018).

[4] CAAM (2020). Economic Performance of the Automobile Industry in 2019. http://www.caam.org.cn/chn/1/cate_3/con_5228367.html.

[5] Lv Z., Chu A.M., McAleer M., Wong W.K. (2019). Modelling economic growth, carbon emissions, and fossil fuel consumption in china: Cointegration and multivariate causality. Int. J. Environ. Res. Public Health.

[6] Peng Y., Dong X. (2017). China Oil and Gas Industry Development Analysis and Prospect Report (2016–2017).

[7] Ma C., Madaniyazi L., Xie Y. (2021). Impact of the Electric Vehicle Policies on Environment and Health in the Beijing–Tianjin–Hebei Region. Int. J. Environ. Res. Public Health.

[8] ETRI (2020). Oil and Gas Industry Development Report at Home and Abroad in 2019.

[9] IEA World Energy Outlook Special Report 2015: Energy and Climate Change. https://webstore.iea.org/weo-2015-special-report-energy-and-climate-change.

[10] National Development and Reform Commission (2015). China Submitted Its Intended Nationally Determined Contribution to Climate Change. http://www.ndrc.gov.cn/xwzx/xwfb/201506/t20150630_710204.html.

[11] MEE (2020). President Xi’s Speech at the General Debate of the 75th Session of the United Nations General Assembly. https://www.mee.gov.cn/ywdt/szyw/202009/t20200923_799945.shtml.

[12] Standardization Administration of China (2004). GB 19578-2004. Fuel Consumption Limits for Passenger Cars.

[13] Standardization Administration of China (2011). GB 27999-2011. Fuel Consumption Evaluation Methods and Targets for Passenger Cars.

[14] Standardization Administration of China (2014). GB 19578-2014. Fuel Consumption Limits for Passenger Cars.

[15] Standardization Administration of China (2014). GB 27999-2014. Fuel Consumption Evaluation Methods and Targets for Passenger Cars.

[16] iCET (2016). Annual Report on Passenger Car Corporate Average Fuel Consumption in China (2016).

[17] Standardization Administration of China (2019). GB 19578-2019. Fuel Consumption Limits for Passenger Cars.

[18] Standardization Administration of China (2019). GB 27999-2019. Fuel Consumption Evaluation Methods and Targets for Passenger Cars.

[19] Ministry of Science and Technology of the People’s Republic of China (2017). Medium and Long-Term Development Plan of Automobile Industry. http://www.most.gov.cn/tztg/201705/t20170510_132694.htm.

[20] The State Council (2020). Development Plan of New Energy Vehicle Industry (2021–2035). http://www.gov.cn/zhengce/content/2020-11/02/content_5556716.htm.

[21] Pavlovic J., Ciuffo B., Fontaras G., Valverde V., Marotta A. (2018). How much difference in type-approval CO2 emissions from passenger cars in Europe can be expected from changing to the new test procedure (NEDC vs. WLTP)?. Transp. Res. Part A Policy Pract..

[22] Ministry of Ecology and Environment (2013). GB 18352.5-2013. Limits and Measurement Methods for Emissions from Light-Duty Vehicles (CHINA 5).

[23] Ministry of Ecology and Environment (2016). GB 18352.6-2016. Limits and Measurement Methods for Emissions from Light-Duty Vehicles (CHINA 6).

[24] Marotta A., Pavlovic J., Ciuffo B., Serra S., Fontaras G. (2015). Gaseous emissions from light-duty vehicles: Moving from NEDC to the new WLTP test procedure. Environ. Sci. Technol..

[25] Mock P., Kühlwein J., Tietge U., Franco V., Bandivadekar A., German J. (2014). The WLTP: How a new test procedure for cars will affect fuel consumption values in the EU. Int. Counc. Clean Transp..

[26] Schmidt H. (2015). Worldwide harmonized light-vehicles test procedure (wltp) und real driving emissions (rde)–aktueller stand der diskussion und erste messergebnisse. 15. Internationales Stuttgarter Symposium.

[27] Liu X., Zhao F., Hao H., Chen K., Liu Z., Babiker H., Amer A.A. (2020). From NEDC to WLTP: Effect on the Energy Consumption, NEV Credits, and Subsidies Policies of PHEV in the Chinese Market. Sustainability.

[28] Pavlovic J., Marotta A., Ciuffo B. (2016). CO2 emissions and energy demands of vehicles tested under the NEDC and the new WLTP type approval test procedures. Appl. Energy.

[29] Tsiakmakis S., Fontaras G., Cubito C., Pavlovic J., Anagnostopoulos K., Ciuffo B. (2017). From Nedc to Wltp: Effect on the Type-Approval co2 Emissions of Light-Duty Vehicles.

[30] Tsiakmakis S., Fontaras G., Ciuffo B., Samaras Z. (2017). A simulation-based methodology for quantifying European passenger car fleet CO2 emissions. Appl. Energy.

[31] Hill N., Windisch E., Kirsch F., Horton G., Dun C., Energy R., Hausberger S. (2016). Improving Understanding of Technology and Costs for CO2 Reductions from Cars and LCVs in the Period to 2030 and Development of Cost Curves.

[32] Krause J., Donati A.V., Thiel C. (2017). Light Duty Vehicle CO2 Emission Reduction Cost Curves and Cost Assessment-the DIONE Model.

[33] Lipman T.E. (2017). Emerging technologies for higher fuel economy automobile standards. Annu. Rev. Environ. Resour..

[34] National Research Council (2015). Cost, Effectiveness, and Deployment of Fuel Economy Technologies for Light-Duty Vehicles.

[35] ICCT (2017). 2017 Global Update: Light-Duty Vehicle Greenhouse Gas and Fuel Economy Standards. https://theicct.org/publications/2017-global-update-LDV-GHG-FE-standards.

[36] CPCA (2020). Statistics of China’s Passenger Car Market. http://data.cpcaauto.com/SegmentMarket.

[37] CPCA (2016). Statistics of China’s Passenger Car Market. http://www.cpcaauto.com/newslist.asp?types=csjd&id=6213.

[38] Wang S., Zhao F., Liu Z., Hao H. (2017). Heuristic method for automakers’ technological strategy making towards fuel economy regulations based on genetic algorithm: A China’s case under corporate average fuel consumption regulation. Appl. Energy.

[39] Wang S., Chen K., Zhao F., Hao H. (2019). Technology pathways for complying with Corporate Average Fuel Consumption regulations up to 2030: A case study of China. Appl. Energy.

[40] Chen K., Zhao F., Liu Z., Hao H. (2019). Technology routes to meet China’s future corporate average fuel consumption regulations. Automob. Technol..

[41] SAE-China (2020). Technology Roadmap for Energy Saving and New Energy Vehicles 2.0.

[42] iCET (2018). Annual Report on Passenger Car Corporate Average Fuel Consumption in China (2017).

[43] iCET (2019). China Passenger Vehicle CAFC-NEV Credits Research Report (2018).

[44] MIIT (2019). Passenger Car Corporate Average Fuel Consumption and New Energy Vehicle Credits in China (2019). https://www.miit.gov.cn/jgsj/zbys/qcgy/art/2020/art_902b6e3db65944dfa8d0ffe61adbce9c.html.

[45] Liu Z. (2017). Zhao Fuquan’s Insights on the Automotive Industry.

[46] Chen K., Zhao F., Liu Z., Hao H. (2018). Fuel Economy Regulations and Technology Roadmaps of China and the US: Comparison and Outlook (No. 2018-01-1826).

[47] Wang Y., Zhao F., Yuan Y., Hao H., Liu Z. (2018). Analysis of typical automakers’ strategies for meeting the dual-credit regulations regarding Cafc and Nevs. Automot. Innov..

[48] He H., Bandivadekar A. (2013). Passenger Car Fuel-Efficiency, 2020–2025 Comparing Stringency and Technology Feasibility of the Chinese and US Standards.

[49] National Technical Committee of Auto Standardization (2019). GB/T 38146.1-2019. Driving Cycle of the Chinese Vehicle Part 1: Light-Duty Vehicles.

